# An Analysis of Resting-State Functional Transcranial Doppler Recordings from Middle Cerebral Arteries

**DOI:** 10.1371/journal.pone.0055405

**Published:** 2013-02-06

**Authors:** Ervin Sejdić, Dmitry Kalika, Nicholas Czarnek

**Affiliations:** Department of Electrical and Computer Engineering, Swanson School of Engineering, University of Pittsburgh, Pittsburgh, Pennsylvania, United States of America; Charité University Medicine Berlin, Germany

## Abstract

Functional transcrannial Doppler (fTCD) is used for monitoring the hemodynamics characteristics of major cerebral arteries. Its resting-state characteristics are known only when considering the maximal velocity corresponding to the highest Doppler shift (so called the envelope signals). Significantly more information about the resting-state fTCD can be gained when considering the raw cerebral blood flow velocity (CBFV) recordings. In this paper, we considered simultaneously acquired envelope and raw CBFV signals. Specifically, we collected bilateral CBFV recordings from left and right middle cerebral arteries using 20 healthy subjects (10 females). The data collection lasted for 15 minutes. The subjects were asked to remain awake, stay silent, and try to remain thought-free during the data collection. Time, frequency and time-frequency features were extracted from both the raw and the envelope CBFV signals. The effects of age, sex and body-mass index were examined on the extracted features. The results showed that the raw CBFV signals had a higher frequency content, and its temporal structures were almost uncorrelated. The information-theoretic features showed that the raw recordings from left and right middle cerebral arteries had higher content of mutual information than the envelope signals. Age and body-mass index did not have statistically significant effects on the extracted features. Sex-based differences were observed in all three domains and for both, the envelope signals and the raw CBFV signals. These findings indicate that the raw CBFV signals provide valuable information about the cerebral blood flow which can be utilized in further validation of fTCD as a clinical tool.

## Introduction

Cerebral metabolism and brain function are related to cerebral blood flow [1]. Various imaging modalities such as functional magnetic resonance imaging (e.g., [2]) were used to demonstrate that mental and motor activities result in an increased cerebral blood flow in the feeding bed arteries, especially because of increased regional demand for 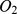, glucose and other metabolites [1]. However, these imaging techniques can be expensive and complex to operate [3], and functional transcranial Doppler (fTCD) arose as a viable alternative to monitor cerebral blood flow. fTCD is a noninvasive and easily operated ultrasound diagnostic technique that can be used to monitor the hemodynamic characteristics of major cerebral arteries in normal and pathologic conditions [4]. Measurements are taken with a probe placed on the skull of a subject, usually over the transtemporal window [5], which enables the monitoring of main cerebral arteries. fTCD has a high temporal resolution and has been used in many psychophysiologic studies involving various cognitive tasks (e.g., [3], [5], [6], [7], [8], [9]). These studies demonstrated that mean cerebral blood flow velocity (CBFV) obtained from fTCD data increases when users are doing a cognitive activity compared with baseline periods [10], [11], [12], [13].

**Figure 1:**
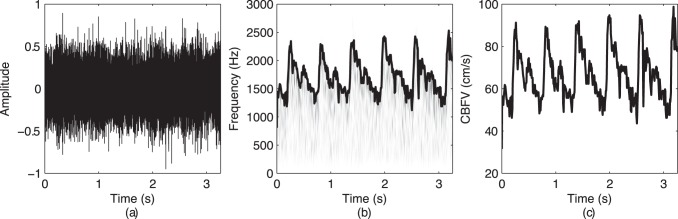
Relationship between the raw CBFV signal and its maximal velocity: (a) a sample raw CBFV signal; (b) its time-frequency representation with the thick black line denoting the maximal velocity (i.e., the envelope signal); (c) the envelope signal converted to cm/s.

The resting-state imaging, especially resting-state functional magnetic resonance imaging, arose in recent years as a method to investigate the regional interactions when a subject is not performing an explicit task. Therefore, it is important to characterize the resting-state CBFV using fTCD as the resting-state activity of the brain can influence the consequent mental tasks. Early studies considering the resting-state fTCD showed that the measured CBFV at rest decreased significantly with increasing age [14], [15], [16]. Also, younger females had significantly higher blood velocity values than males [14], [15], [16]. However, previous publications dominantly considered the maximal velocity corresponding to the highest Doppler shift as a measure of the CBFV (e.g., [3]–[17]). Figure 1 depicts a relationship between the raw CBFV signal as captured by fTCD and the maximal velocity, which is usually called the spectral envelope signal. It is clear that the envelope signal only partly captures information about cerebral blood flow, as the received ultrasound signal is a sum of sinusoidal components corresponding to a large number of blood particles moving at different velocities [17]. Therefore, even though the resting-state values for fTCD have been previously investigated, there is a lack of knowledge to date regarding the relationship between the raw CBFV signals and the envelope signals and what additional information can be gained from the raw CBFV signals. This is a particularly important issue as fTCD is becoming an important clinical tool to study cerebral blood flow and was a recently proposed as a viable option for brain-to-computer interfaces [18].

There are two major contributions of the current study. First, the characterization of the resting-state fTCD using simultaneously acquired raw and envelope CBFV signals is novel, as previous studies focused only on the envelope signals. Second, the paper introduces several analysis approaches such as information-theoretic approaches and time-frequency techniques to analyze these resting-state signals. Most of the previous contributions focused on statistical analysis of the envelope signals, and some recent contributions considered more advanced tools such as frequency analysis (e.g., [9]).

## Methods

### Data Acquisition

Twenty able-bodied participants were recruited, screened, and tested (10 females; 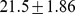 years old (19–26 years old); 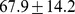 kg (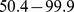 kg); 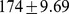 cm (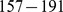) cm; body mass index: 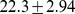 (17.7–29.1)). No participants had a history of heart murmurs, strokes, concussions, migraines, or other brain conditions. At the beginning of the experiment session, each participant was seated in front of a desk and a computer monitor. The participants filled out screening questionnaires, Edinburgh handedness tests, and consent forms which were all approved by the University of Pittsburgh Institutional Review Board. The entire testing procedure was explained to each participant prior to data acquisition. Handedness was determined using the Edinburgh handedness test [19]. 16 subjects were right handed with a mean score of 64% and a range of 38–93%, while three subjects were left handed with a mean score of 80% and a range of 76% to 88%. For one subject, it has been determined that the subject equally uses right and left hands.

A SONARA TCD system (CareFusion, San Diego, CA, USA) was used to acquire CBFV data from the middle cerebral arteries (MCA). Two 2 MHz transducers were used simultaneously to gather bilateral CBFV measurements from the left MCA (L-MCA) and the right MCA (R-MCA). The fTCD transducers were placed at the subjects' transorbital windows on both the left and right side of the skull. The transorbital window lies above the zygomatic arch, located 1–5 cm in front of the ear [6]. The locations, angles, and insonation depth of the TCD transducer were modified until a correct MCA flow was found. The procedure to find the area of insonation was based on Alexandrov et al. [20]. Upon finding the area of insonation, the two TCD transducers were affixed to the participant using a headset. As changes in end-tidal CO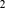 (ETCO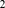) levels are known to affect CBFV in the MCA [21], a Capnocheck Sleep Capnograph/Oximeter (Smiths Medical, Dublin, OH, USA) was used to monitor ETCO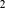. Subjects wore nasal cannulas that were placed after the TCD transducers were affixed.

The participants were instructed to remain awake, stay silent, and try to remain thought-free throughout the 15-minute resting period. Upon the completion the 15-minute resting period, the participants were asked to complete other tasks, which are not considered in the current study. The depth of insonnation for both L-MCA and R-MCA was 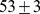 mm.

Once the data acquisition was completed, data were exported using the fTCD system's custom software. The data were extracted as audio files sampled at 44100 Hz representing the cerebral blood flow from R-MCA and L-MCA. The data were downsampled to 8820 Hz in order to speed up the calculations.

### Feature Extraction

Considering a general form of a signal 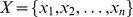, where 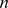 is the total number of samples the following statistical features were extracted:

- Standard deviation of the signal amplitude is related to the AC signal power and typically measures the spread of the amplitude distribution [22]:
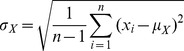
(1)where 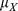 denotes the mean of the signal.
- The skewness of the amplitude distribution computed as follows [22]:
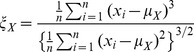
(2)measures the asymmetry of the distribution.
- The kurtosis of the amplitude distribution computed as follows [22]:
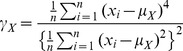
(3)measures how the distribution behaves near the extremes (i.e., whether or not it decays slowly).
- The cross-correlation coefficient at the zeroth lag between 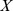 and 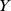 was computed as follows [22]:
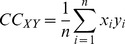
(4)and it assesses the similarity of time structures of 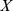 and 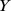.

Next, we consider information-theoretic features often used in the analysis of biomedical signals (e.g., [23], [24], [25]):

- The Lempel-Ziv complexity (LZC) measures the predictability of the signal [25], [26]. To compute LZC, the signal needs to be first converted to a sequence of finite symbols which was achieved with 99 thresholds, 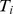, 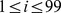, 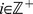. This inherently divides the signal amplitude distribution into 100 equal spaces (i.e. 100 symbols). Next, the quantized signal 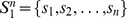 was decomposed into 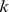 blocks. A block is a sequence of consecutive symbols of length 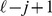 which can be expressed as follows:

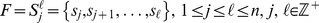
(5)The first block was simply initialized to be the first symbol, i.e. and subsequent blocks were determined based on the following relation.
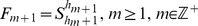
(6)where 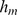 is the ending index for 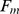, such that 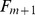 is a unique sequence of minimal length in the sequence 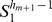. Finally, LZC was computed as follows:
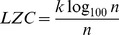
(7)where the logarithmic base of 100 was used because the signal was quantized to 100 symbols.

- The entropy rate (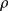) measure quantifies the extent of regularity in a signal [24]. The measure is particularly useful when a relationship among consecutive data points is anticipated. The first step is to to normalize 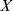 to zero mean and unit variance, by subtracting 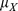 and dividing by 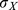. The normalized 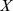 was then quantized into 10 equally spaced levels represented by integers from 0 to 9, ranging from the minimum to maximum value. Using the quantized signal, 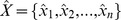, sequences of consecutive points in 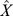 of length 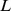, 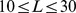, were coded as a series of integers, 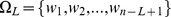, according to the following:

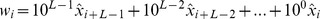
.

This implies that 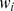 ranged from 0 to 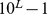 and base 10 was used because there were 10 quantization levels. The Shannon entropy of 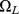 was defined as follows:
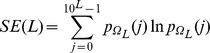
where 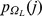 represents the probability of the value j in 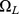, approximated by the corresponding sample frequency. The normalized entropy rate was then computed as follows:



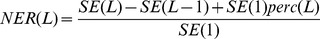
where 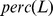 is the percentage of the coded integers in 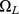 that occurred only once. Finally, an index of regularity, 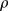, was calculated as the entropy rate feature in this study:



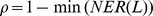
which ranged from 0 (maximum randomness) to 1 (maximum regularity).

- Extending the entropy rate measure, the cross-entropy rate (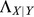) quantifies the entropy rate between two stochastic processes [23]. This measure describes the predictability of a data point in one signal given a sequence of current and past data points in the other signal. First, both 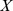 and 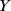 were normalized, quantized, and coded using the same methodology as for the entropy rate feature, yielding 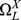 and 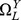, respectively, with 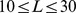. In addition, 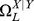 was constructed as follows:

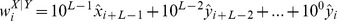
where 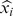 and 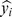 are the quantized samples of X and Y. Then, with 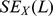, 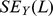, and 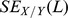 representing the Shannon entropies of 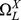, 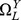, and 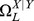, respectively, the normalized cross-entropy of X given Y was computed as follows:



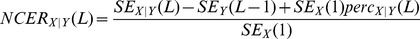
where 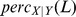 is the percentage of the elements in 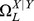 that occurred only once. Next, the uncoupling function was defined as follows:



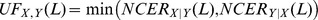



Finally, the following index of synchronization was computed and utilized as the cross-entropy rate feature in this study:
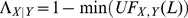
(8)which ranged from 0 (X and Y are completely uncoupled) to 1 (perfect synchronization).

In the frequency domain, we consider the following three features [27]:

- The peak frequency associated with the maximum spectral power was determined by the following:
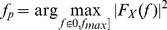
(9)where 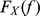 is the Fourier transform of the signal 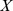 and 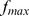 in this study was 4410 Hz.
- The centroid frequency was computed as follows:
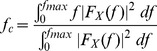
(10)
- The bandwidth of the spectrum was defined as follows:

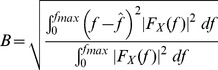
(11)Lastly, we consider typically used time-frequency features (e.g., [28], [29]):

- The relative energy in different time-frequency bands was calculated based on the wavelet transform. A 10-level discrete wavelet decomposition of the signal using the discrete Meyer wavelet was used (e.g. [28], [29]). The resulting decomposition is given by 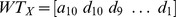, where 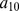 are the approximation coefficients and 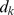 is the 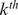-level detail coefficients. Then, the relative energy from the approximation coefficients can be computed as follows:
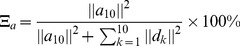
(12)where 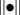 is the Euclidean norm. Using the detail coefficients at the 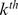-level, the relative energy for that level can be obtained as:
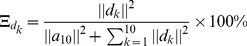
(13)
- A wavelet entropy given by [29]:
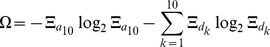
(14)measures the degree of time-frequency based order-disorder of the signal. In other words, more complex time-frequency structures (e.g., a linear FM signal) will have a higher value of 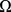 than those structures represented for a simple sinusoidal signal.

### Statistical Tests

To test for statistical differences on the extracted features, we used non-parametric tests such as the Kruskal-Wallis (e.g., [30]) and the Mann-Whitney test (e.g., [31]) test. We also used a linear regression test to examine any dependence between the extracted features and age/BMI, with the null hypothesis being that there is no linear relationship (i.e., the slope is zero).

## Results

The next few subsections summarize the results of the analysis. The results are grouped according to the type of the feature. It should be mentioned that none of the extracted features were affected by age or BMI (linear regression test, 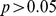). Also, none of the participants experienced great variations in ETCO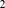 values (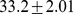 mmHg) and the values were not affected by age or BMI (linear regression test, 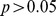).

### Statistical Features

**Figure 2:**
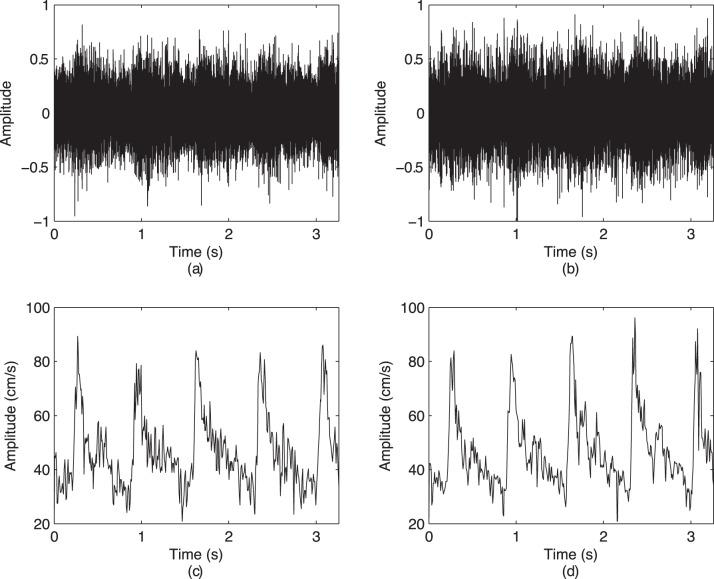
Comparison of sample raw signals and the envelope signals: (a) the raw CBFV signal from the right MCA; (b) the raw CBFV signal from the left MCA; (c) the envelope CBFV signal from the right MCA; (d) the envelope CBFV signal from the left MCA.

Table 1 summarizes the results for statistical features. There were no statistical differences for any of the parameters between left and right sides for either the raw signals or for the envelope signals (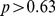). However, it can be observed that the raw CBFV signals and the envelope CBFV signals have completely different statistical characteristics due to their appearances in the time domain (please see Figure 2).

**Table 1:** A summary of statistical features extracted from the raw and envelope CBFV signals.

	Raw		Envelope	
	L-MCA	R-MCA	L-MCA	R-MCA
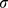	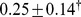	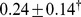	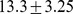	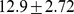
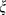	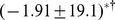	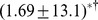	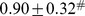	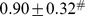
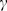	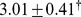	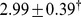	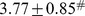	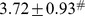
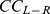	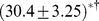		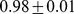	

An asterisk denotes multiplication by 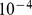.

† statistical differences between the envolope- and raw signal based features.

# sex-based differences.

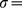
 standard deviation; 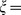 skewness; 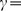 kurtosis; 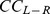 = cross-correlation coefficient.

The envelope signals had a greater spread of amplitude values as observed differences in values of standard deviations (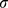). The skewness (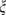) values also showed that the amplitude distributions for the envelope were more to the right side from their mean values in comparison to the raw CBFV signal. This was reinforced by the kurtosis values (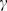) which showed that the amplitude distributions for the envelope signals had more extreme values in comparison to the amplitude distributions for the raw CBFV signals. Furthermore, the skewness (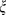) and the kurtosis (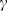) on both sides were affected by sex when considering the envelope CBFV signals (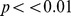). In particular, when considering R-MCA, 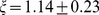 and 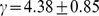 for males, and 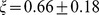 and 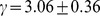 for females. Similarly, when considering L-MCA, 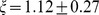 and 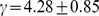 for males, and 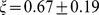 and 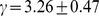 for females. However, sex did not impose any statistical differences on these features from the left or right MCA for the raw CBFV signals. The most striking difference is almost zero correlation between the raw CBFV signals from the left and right MCAs. On the other hand, the envelope signals were very highly correlated.

### Information-theoretic Features

The information-theoretic features for the considered signals are shown in Table 2. There were no statistical differences between left and right sides for either the raw or envelope CBFV signals (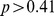).

**Table 2:** Information-theoretic features extracted from the collected CBFV signals.

	Raw		Envelope	
	L-MCA	R-MCA	L-MCA	R-MCA
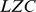	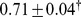	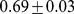	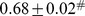	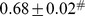
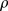	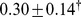	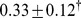	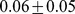	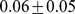
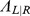	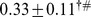		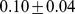	

† statistical differences between the envolope- and raw signal based features.

# sex-based differences.

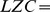
 Lempel-Ziv complexity; 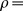 entropy rate; 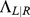 = synchronization index.

The predictability of the signal as measured by LZC showed that the raw and the envelope signals from R-MCA were not statistically different (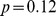), while LZC from L-MCA were statistically different for the raw and the envelope signals. The raw CBFV signals from L-MCA were less predictable than the signals from L-MCA based on the envelope signals. Furthermore, LZC was influenced by sex for both left (females: 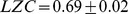; males: 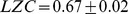; 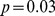) and right (females: 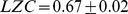; males: 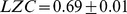; 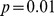) sides when considering the envelope CBFV signals.

When considering the regularity of the signals as measured by 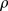, it was interesting to note that the raw CBFV signals had statistically higher regularity than the envelope CBFV signals (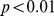). In other words, the consecutive data points in the raw CBFV signals were more related to each other than the consecutive points in the envelope signals. In fact, the envelope CBFV signals are almost random given the very low 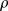 values.

The synchronization index, 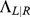, was statistically higher for the raw CBFV signals than for the envelope signals (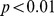). That is, the left and right raw CBFV signals have more common information than the left and right envelope signals. However, there were sex-based differences for the raw signals (females: 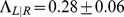; males: 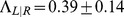; 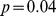).

### Frequency Features

**Figure 3:**
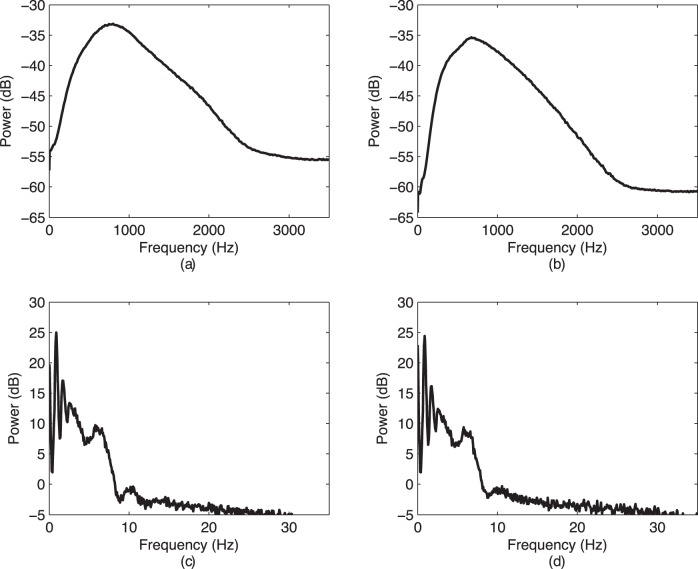
Spectral content of the raw and envelope signals from a single subject obtained through estimates of power spectral densities (PSD) using the Welch method: (a) a PSD of a raw CBFV from R-MCA; (b) a PSD of a raw CBFV from L-MCA; (c) a PSD of the envelope CBFV from R-MCA; and (d) a PSD of the envelope CBFV from L-MCA.

The considered spectral features are summarized in Table 3, while the power spectral densities of the raw and envelope signals from both sides are depicted in Figure 3. No statistical differences were observed between the features stemming from the left and right MCA (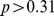). The features stemming from the raw CBFV signals had statistically higher values than the features based on the envelope signals (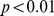). Interestingly, when considering the raw CBFV signals from R-MCA, the spectral centroid, 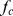, was statistically different between males (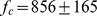 Hz) and females (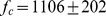 Hz) (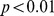). Similarly, there were sex-based statistical differences on the peak frequency, 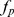 (females: 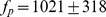 Hz; males: 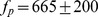 Hz; 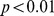). The recordings from L-MCA or based on the envelope CBFV signals were not affected by sex.

**Table 3:** Frequency characteristics considered in this study.

	Raw		Envelope	
	L-MCA	R-MCA	L-MCA	R-MCA
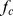 (Hz)	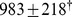	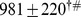	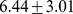	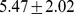
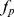 (Hz)	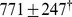	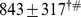	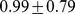	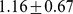
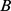 (Hz)	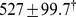	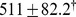	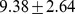	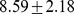

† statistical differences between the envolope- and raw signal based features.

# sex-based differences.

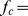
 spectral centroid; 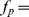 peak frequency; 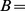 bandwidth.

### Time-frequency Features

**Figure 4:**
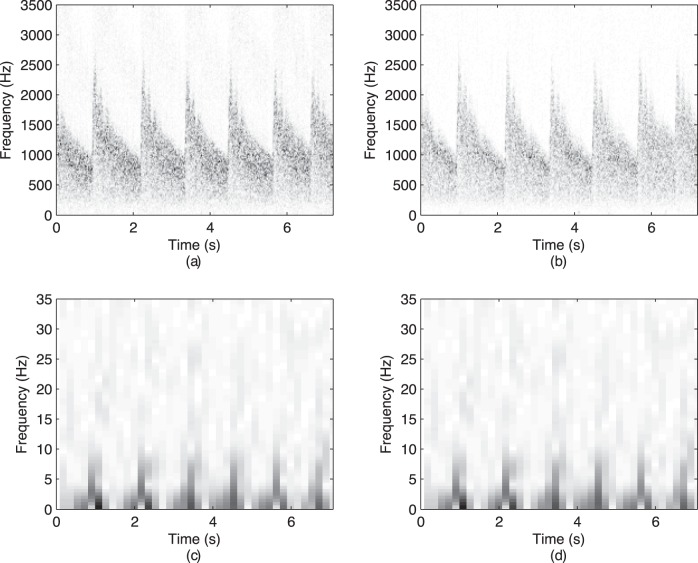
Time-frequency representations (TFR) of the raw and the envelope signals form a single subject: (a) a TFR of a raw CBFV from R-MCA; (b) a TFR of a raw CBFV from L-MCA; (c) a TFR of the envelope CBFV from R-MCA; and (d) a TFR of the envelope CBFV from L-MCA.

The time-frequency features are summarized in Table 4. Time-frequency representations of a few seconds of sample raw and envelope signals are shown in Figure 4. The wavelet entropy, 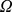, was statistically higher for the raw signals than for the envelope signals. Additionally, there were sex-based differences (females: 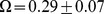; males: 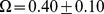) in 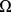 for the envelope signals from R-MCA (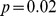). When considering the distribution of energy in different time-frequency bands, it can be noticed that the envelope signals have almost all of their energy concentrated in a very low-frequency region, while the raw CBFV signals have most of their energy concentrated in higher frequency bands. The energy concentration in the low-frequency region for the envelope signal from R-MCA was affected by sex (females: 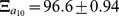; males: 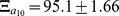; 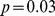). On the other hand, the raw CBFV signals are mostly concentrated (around 99% of energy) in the higher-frequency regions (from 6th to 10th decomposition levels). Similarly as for the envelope signals, the energy concentration for the raw CBFV signals from R-MCA was affected by sex. In particular, the following features were affected: 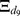 (females: 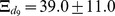; males: 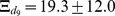; 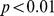), 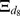 (females: 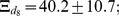 males: 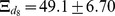; 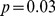) and 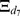 (females: 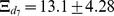; males: 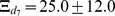; 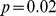).

**Table 4:** Time-frequency features extracted from the collected signals.

	Raw		Envelope	
	L-MCA	R-MCA	L-MCA	R-MCA
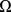	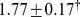	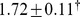	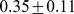	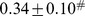
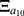	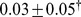	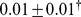	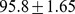	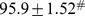
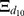	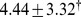	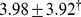	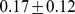	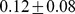
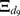	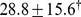	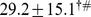	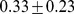	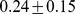
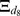	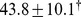	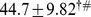	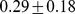	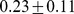
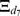	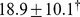	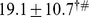	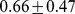	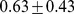
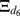	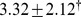	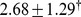	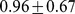	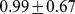
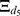	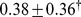	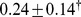	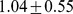	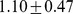
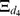	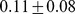	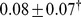	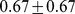	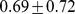
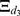	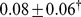	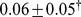	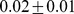	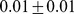
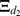	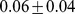	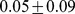	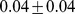	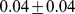
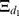	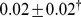	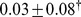	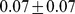	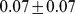

† statistical differences between the envolope- and raw signal based features.

# sex-based differences.

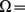
 wavelet entropy; 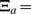 the relative energy from from the approximation coefficients; 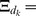 the relative energy from the detail coefficients at the 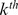 level.

## Discussion

The statistical analysis of the resting-state fTCD signals yielded interesting results. First, the statistical features considered here were greatly affected by sex when considering the envelope CBFV signals. In particular, the shape of probability distributions of amplitudes were affected by sex as shown by sex-based effects on kurtosis and skewness. On the other hand, sex did not play any significant role while examining these statistical features from the raw CBFV signals. Second, while the envelope-based signals were greatly correlated, the raw CBFV recordings from two L-MCA and R-MCA exhibited very low correlation. In other words, the signals from these two arteries have very different temporal structures. However, interestingly enough, none of the considered features were statistically different between left and right sides. This is a very important finding when considering the applications of fTCD such as cerebral laterilazation during different cognitive tasks (e.g., language tasks considered in [32]).

The information-theoretic analysis of the recordings showed that both the raw and the envelope CBFV signals have almost identical predictability as measured by LZC. However, LZCs were statistically different for recordings from L-MCA, which were still around 0.7. This indicates that the signals from the both groups are equally complex. That is, the sources producing both groups of signals have equal complexity, which in our case, it is an identical source. However, when considering the entropy rate (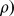, we found that the envelope CBFV signals have very low entropy rate. In fact, their entropy rates are very close to zero, which indicates a random process. On the other hand, the entropy rates for the raw CBFV signals were significantly higher due to the fact that raw CBFV signals are composed of multiple weighted sinusoid signals (i.e., a pure single-frequency sinusoid signal can have very high value for the entropy rate). Additionally, the raw CBFV signals also had higher values for the synchronization index, 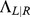. From a physiological point of view, it means that the raw recordings from L-MCA and R-MCA contained more mutual information than the envelope signals. This is a very important finding as the temporal structures of these raw signals are very different as demonstrated by very low correlation between them. On the other hand, it was interesting to observe that the envelope CBFV signals had almost identical temporal structure, but the contained mutual information is significantly lower than for the raw CBFV signals. Lastly, male participants had higher 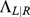 values than the female participants when considering the raw CBFV signals. However, these differences may be due to the limited number of subjects as 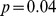.

The frequency analysis of the recorded signals showed that the raw CBFV signals had a significantly higher frequency content than the envelope CBFV signals. While the envelope CBFV signals are typically low-frequency signals, the raw CBFV signals are usually concentrated around frequencies close to 1000 Hz. Therefore, by analyzing the raw CBFV signals, we may avoid considering the very low-frequency physiological artifacts that can impact the envelope CBFV signals and avoid any pre-processing of these signals (e.g., [33]). Furthermore, we observed that females had a higher spectral content as measured by the peak and central frequency for the raw CBFV recordings from R-MCA, but not from L-MCA. This resonates with [34], which found that females had a slightly stronger flow in R-MCA than males. However, this frequency shift was not observed for L-MCA or when using the envelope CBFV signals from either sides.

The time-frequency analysis of the recorded signals showed that the raw CBFV signals have significantly higher values for the wavelet entropies than the envelope CBFV signals. This signifies that the time-frequency structure of the envelope signals is simpler and more ordered than the time-frequency structure of the raw CBFV signals. This is clear from the energy concentration for different bands shown in Table 4. The envelope signals have most of their energy concentrated in the the low-frequency band, whereas the raw signals have 99% of their energy concentrated across several higher frequency bands. Furthermore, the sex-differences observed in the frequency content of the raw recordings from R-MCA can be also noticed in the several time-frequency bands. These findings indicated that any changes in the time-frequency content are actually due to the changes in the spectral content of signals.

Lastly, the presented results demonstrated that the raw CBFV signals should be considered in addition to the envelope CBFV signals typically analyzed in many contributions (e.g., [3], [5], [6], [7], [8], [9]) as these signals have completely different time, frequency and time-frequency characteristics and provide a rich source of information. Such information may be very valuable in the further development of brain-to-computer interfaces or other clinical applications of fTCD.

### Conclusions

In this article, we characterized the resting-state fTCD using the simultaneously acquired raw and envelope CBFV signals. Time, frequency and time-frequency features were extracted from these signals and compared. Specifically, the time structures of recordings from L-MCA and R-MCA were strongly correlated when considering the envelope signals, but had very low correlation when considering the raw signals. On the other hand, the raw signals had a higher level of mutual information in comparison to the envelope signals. Frequency characteristics were also different. We showed that the raw CBFV signals were centered around 1000 Hz, while the envelope signals were centered around 1 Hz. The raw signals also had their energy concentration across several time-frequency bands, while the envelope signals had strong energy concentration in the low-frequency region (i.e., fully described by the approximation coefficients of the wavelet transform). These results showed that the raw CBFV signals have very different signal characteristics and can contribute valuable information regarding cerebral blood flow.
